# Relationship between Emotional Intelligence, Sleep Quality and Body Mass Index in Emergency Nurses

**DOI:** 10.3390/healthcare9050607

**Published:** 2021-05-18

**Authors:** Noelia Miguez-Torres, Alejandro Martínez-Rodríguez, María Martínez-Olcina, Laura Miralles-Amorós, Cristina Reche-García

**Affiliations:** 1 Department of Nursing, Faculty of Nursing, Catholic University of Murcia, 30107 Murcia, Spain; nmiguez@alu.ucam.edu (N.M.-T.); creche@ucam.edu (C.R.-G.); 2 Department of Analytical Chemistry, Nutrition and Food Sciences, Faculty of Sciences, University of Alicante, 03690 Alicante, Spain; maria.martinezolcina@ua.es (M.M.-O.); lma52@alu.ua.es (L.M.-A.); 3 Alicante Institute for Health and Biomedical Research (ISABIAL Foundation), 03010 Alicante, Spain

**Keywords:** emotional intelligence, nursing, emergencies, quality of sleep, quality of life, health care and services

## Abstract

Nurses have long working hours with high psychological burdens. In addition, in the emergency department, nurses are required to quickly adapt emotionally. The aim of this study was to describe and relate emotional intelligence (EI) skills of emergency nurses, their body mass index (BMI) and sleep quality. For this purpose, a cross-sectional was carried out in which the perceived emotional intelligence test and the Pittsburgh sleep quality index were applied. Sixty-two emergency nurses (48 women and 14 men) participated. The results indicated that the majority of them present adequate levels of EI, with no differences by gender. Younger nurses showed a better ability to feel, express and understand emotional states than the older ones, while the ability to regulate emotional states occurred in the opposite way. Nurses who have been working for several years showed a better ability to regulate emotions than those with less experience. Those who were overweight grade II and obese type I expressed their feelings better, also the regulation of emotional states decreased as weight increased. Finally, it has been observed that the quality of sleep of emergency nurses is significantly altered, and that this lack of sleep may affect their ability to process emotions.

## 1. Introduction

Emergency health professionals are constantly faced with urgent situations and high-risk interventions, which frequently leads to burnout syndrome [1,2]. Burnout syndrome has often been linked to emotional intelligence, especially in healthcare staff [3,4]. Emotional intelligence is a significant predictor of low burnout [3,5,6].

Emotional intelligence (EI) is defined as “the subset of social intelligence that involves the ability to monitor one’s own and others’ feelings and emotions, to discriminate among them and to use this information to guide one’s thinking and actions” [7,8,9].

In contrast to this integrative model, there are mixed models [8,9]. The author Bar-On refers to non-cognitive skills and defines EI as a set of skills that a person must cope with the demands and impositions of the environment. He also mentions the extent of personal success, which depends on various aspects of personality [8,9]. His model highlights five important areas for measuring EI: intrapersonal (emotional self-awareness, assertiveness, independence, self-esteem and self-actualization); interpersonal (interpersonal relationships, empathy and social responsibility); adaptability (decisiveness, flexibility and ability to correctly assess reality); stress management (stress tolerance and impulse control); and general mood (positivity, happiness, joy and optimism) [10,11].

Nurse’s work shifts of 8, 12 or 24 h can produce high psychological stress [12]. In the emergency department, moreover, rapid emotional adaptation is required of nurses [13,14]. Research suggests that these emergency healthcare workers have greater capacity for emotional self-regulation, and that at higher levels of EI more awareness of feelings, increasing positivity and improving mental health [8,10,15,16].

It has been seen that female nurses tend to pay more attention to emotions, while male nurses are more systematic [17,18]. This not only occurs in that profession, but several studies have observed that the female gender presents better results in the evaluation of EI than men [19,20].

In addition, work experience, academic studies and age also seem to influence the level of EI [21,22]. However, some studies differ from this relationship and explain that the most significant age for the development of appropriate EI is mainly childhood. This excludes the relationship between the development of emotions and higher academic and/or university education [23].

It is known that nurses routinely suffer alterations in sleep quality, not only due to work schedules, also because of psychological stress [24,25,26]. Sleeping few hours per day for a prolonged period of time is associated with an alteration of the brain mechanism and its function in the emotional field, resulting in a more negative mood (behavioral impairment) [11,27,28,29]. Lack of sleep has been found to impair abilities that have a specific role in EI and may affect emotion processing [28,30]. Surani et al. also report that lack of sleep affects work performance, increasing the probability of making mistakes [11,31,32]. A secondary problem to poor sleep quality could be when the lack of emotional management is masked and accumulates and presents itself, for example, in the form of nightmares [28,33,34].

Another alteration that has been related to EI in nursing personnel is poor nutrition, with high prevalence of overweight and obesity [24,35,36]. It seems that as a person’s weight increases, the level of EI decreases, due to the fact that weight gain is sometimes linked to a lack of emotional self-regulation and ability to express oneself [24,37].

Age and weight have been shown to be parameters to be assessed for negative influences related to physical and psychological factors, such as eating behaviors, insomnia, mood and anxiety [38]. It has been observed that nursing work presents high job stress and psychosocial risks, increasing with work experience [38].

Nowadays, there are not enough data describing the relationship between the emotional intelligence of emergency department nurses and variables such as age, gender and work experience. Moreover, studies linking the emotional intelligence of these professionals with sleep quality and/or body mass index (BMI) are considered to be scarce.

Therefore, the general objective of this study was to describe and relate the perception of EI skills of nurses working in emergency departments, their BMI and sleep quality. The specific objectives are (a) to know if there are differences in the perception of emotional intelligence skills as a function of age, gender and years of experience in the emergency department, and (b) to relate the perception of emotional intelligence skills of emergency nurses to their BMI and sleep quality.

Based on the abovementioned theoretical rationale and the available empirical studies, authors propose the following hypotheses: (1) EI skills are negatively associated with BMI in emergency nurses; (2) EI ability is positively associated with sleep quality in emergency nurses. These hypotheses implicitly assume that autonomous and controlled emotional skills are associated with a healthy lifestyle, based on nutritional status and sleep.

## 2. Materials and Methods

### 2.1. Study Design

A descriptive observational cross-sectional study was conducted. Nurses from Spain were selected, their participation was voluntary. It consisted of an online survey addressed to all nurses who were working at the time of the study or had worked in an emergency department. At the beginning, the volunteers filled out an informed consent form, which they had to accept in order to answer the questions. They were also informed about the reason for the research, thus giving their consent to participate in the study. It was specified that a series of anonymous and confidential questions would be asked. It was performed in accordance with the code of ethics of the World Medical Association (Declaration of Helsinki). This study was approved by the Ethics Committee of the Catholic University of Murcia with code CE022008.

### 2.2. Subjects

The study was conducted between March and April 2020 through an online survey aimed at nurses who have worked in an emergency department in Spain. An email with the survey link was sent to supervisors of emergency departments in Spanish hospitals. Nurses were encouraged to forward the survey link to interested colleagues, seeking a snowball sample of professionals. 

Sixty-two nursing professionals participated. Subjects considered for inclusion were female and male graduates in nursing who work or have worked in an emergency department. All nurses who had never worked in an emergency department were excluded. This study was only in the emergency nursing profession.

### 2.3. Instruments

#### 2.3.1. Sociodemographic and Anthropometry Data

An ad hoc questionnaire was administered asking sociodemographic and employment data. Weight and height measurements were self-reported in this questionnaire by the participants. They were instructed to measure themselves according to the International Society for the Advancement of Kinanthropometry (ISAK) criteria [39]. BMI “weight/(height^2^)” was evaluated following the methodology of the ISAK [39]. It was interpreted using the Spanish Association for the Study of Obesity (SEEDO) classification (“BMI < 18.5 kg/m^2^ underweight”, “BMI between 18.5 to 24.99 kg/m^2^ normal weight”, “BMI between 25 to 26.9 kg/m^2^ overweight grade I”, “BMI between 27 to 29.9 kg/m^2^ overweight grade II (pre-obesity)”, “BMI 30 to 34.9 kg/m^2^ obesity grade I”, “BMI 35 to 39.9 kg/m^2^ obesity grade II” and “BMI 40 to 49.9 kg/m^2^ obesity grade III”) [40]. 

#### 2.3.2. Emotional Intelligence

One of the most widely used questionnaires for the measurement of EI is the “Trait Emotional Meta-Cognition Scale” (TMMS), which is based on the integrative model of Salovey and Mayer [7]. It was developed by Salovey and collaborators in (1995) [7]. Its extended version is constituted by 48 items, and the reduced version is formed by 24 [41]. 

The Trait Meta-Mood Scale (TMMS-24) [42] or Emotional Intelligence Scale translated into Spanish [24] was used. This inventory consists of 24 items that are subdivided into three subscales or dimensions: (a) emotional attention (ability to feel and express feelings); (b) emotional clarity (ability to understand emotions); and (c) emotional repair (ability to regulate emotional states). 

The score for each of these subscales is classified into three ranges. For the emotional perceived subscale, the middle score range (22–32 in male; 25–35 in female) indicates appropriate emotional attention, and scores in the high (>33 in male; >36 in female) or low (<21 in male; <24 in female) range indicate that emotional attention should be improved. In contrast, for the clarity subscale, scores in the low range indicate a need for improvement (<25 in male, <23 in female), those in the middle range (26–35 in male; 24–34 in female) indicate appropriate clarity, and those in the high range (>36 in male; >35 in female) indicate excellent emotional clarity. Likewise, in the emotional repair subscale, low scores (<23 in male and female) indicate the need for improvement, scores in the middle range (24–35 in male, and 24–34 in female) indicate appropriate repair, and high scores (>36 in male, >35 in female) indicate excellent emotional repair. In the questionnaire, individuals must rate each of their responses on a Likert scale from one to five points to indicate their level of agreement. The total score is obtained by adding the responses from each sub-scale, each of which ranges from 8 to 40 points [23,43]. It presents adequate psychometric properties in the Nursing population: Emotional care: Cronbach’s alpha confidence interval CIα = 0.80 (0.77–0.83); Emotional clarity: Ciα = 0.87 (0.85–0.89); Emotional reparation: CIα = 0.85 (0.82–0.87) [44].

#### 2.3.3. Sleep Quality

Pittsburgh Sleep Quality Index (PSQI) developed by Buysse et al. [45] in its Spanish version “Índice de calidad de sueño de Pittsburgh” [46] was used. It has 19 questions with seven areas of measurement: subjective sleep quality, sleep latency, sleep duration, habitual sleep efficiency, sleep disturbances, use of sleeping medication and daytime dysfunction. Each area ranges between 0 and 3 points, with higher scores reflecting greater difficulty. The combined score ranges from 0 (easy sleep) to 21 points (severe difficulty). The Cronbach alpha coefficient for PSQI internal consistency reliability was 0.83 [47]. 

### 2.4. Statistical Analysis

A descriptive analysis was performed for each variable (mean, standard deviation and 95% confidence level). Contingency tables were used to relate the EI scale data to the variables sex, age, time of experience and PSQI; as well as Kolmogorov–Smirnov normality tests, Levene’s test; tests of contrast of means (Student’s *t*-test for sex, and ANOVA and post hoc tests for age and time of work experience, specifically the Games–Howell test, taking into account that equality of variances was not assumed), and Pearson’s correlation (of the PSQI and BMI with EI). The statistical data were processed and analyzed using the IBM SPSS 26.0 statistical software. The level of significance was set at *p* ≤ 0.05.

## 3. Results

Sixty-two nursing assistants participated in the study. The majority were female, BMI 24.2 ± 0.5 kg/m^2^ aged between 21 and 30 years and with 1–5 years of experience. Table 1 shows the general characteristics of the subjects and Table 2 several descriptive characteristics of the main study variables.

After the application of the TMMS-24 scale, it was observed that the majority of nursing professionals working in emergency departments have appropriate levels in the three EI dimensions assessed (Table 3).

To assess sleep quality, the PSQI scale was used to obtain an overall score (0–21 points) and a score for each component (Table 4). The normal assessment is usually made by component and not by total points. Even so, Buysse et al. [45] propose that a score of less than 5 indicates no sleep disturbance, while a score equal to or greater than 5 indicates that there is disturbance. 

It was observed that 66% of the nurses working in the emergency department obtained scores that exceeded the cut-off point of 5, thus presenting sleep problems, while only 34% did not present any sleep disturbance. Likewise, in the individual evaluation of each component, 42% had slight difficulty in “subjective quality of sleep”, 39% in “sleep latency”, 37% in “habitual sleep efficiency”, 65% in “sleep disturbances”, and 39% in “daytime dysfunction”. Forty-four percent have no difficulties in “sleep duration” and 74% in “medication use”.

Contrast tests of means (Student’s t) were performed to relate EI and gender. It has been observed that there are no significant differences in the gender of emergency nursing assistants and their level of EI: emotional attention with a *p* = 0.860, emotional clarity *p* = 0.220 and emotional repair *p* = 0.084. Even so, it can be seen that in both sexes the level of excellent emotional repair is higher than in emotional clarity (Table 5).

Regarding the relationship between emotional intelligence and age, it has been observed that nurses aged between 21 and 30 years and who recently finished their degree have a better level of attention and emotional clarity than nurses with an older age. 

However, when performing the ANOVA test, significant differences were found in the level of emotional repair (*p* = 0.011). Through the Games–Howell test it was seen that there are significant differences between the dependent variable emotional repair and the age ranges. It is observed, therefore, that nurses with a higher age range have a better level of emotional repair than younger nurses in the emergency department.

According to the ANOVA test, there are no significant differences in the level of emotional attention (*p* = 0.746) or in the level of emotional clarity (*p* = 0.827) according to the time of work experience in an emergency department and EI. However, significant differences were found in the level of emotional repair (*p* = 0.021). After performing the Games–Howell test, it was found that there are significant differences between the dependent variable of emotional repair and the ranges of time of experience, concluding that nurses with more experience in an emergency department will have a better emotional repair than those who have been working in that department for only a few months.

In the correlation of data (Pearson) between BMI and appropriate and in appropriate levels of the components of emotional EI, significant data were found only between appropriate emotional attention and grade II overweight (*p* = 0.006) and type I obesity (*p* = 0.042). As weight increases slightly, the level of emotional attention increases. A significant correlation was also seen (*p* = 0.022) between inappropriate emotional repair and type I obesity, i.e., as weight increases, the capacity for emotional repair decreases (Table 6).

Finally, in the data correlation (Pearson) between EI and the seven components of the PSQI, the following significant correlations were found: The longer the duration of sleep, the better the appropriate emotional clarity (*p* = 0.029); the greater the use of medication the less appropriate emotional clarity (*p* = 0.008); as emotional repair worsens, daytime dysfunction increases (*p* = 0.002) (Table 7).

## 4. Discussion

One of the objectives of the present study was to analyze the level of EI of nursing professionals working in emergency departments in Spain, according to age, gender and length of work experience in the emergency department. The findings showed that most of these nurses have appropriate levels in the ability to feel and express feelings (64.5%), in the ability to understand emotions (62.9%) and in the ability to regulate emotional states (67.7%). These data correspond with those found in other studies suggesting that emergency nurses have good emotional self-regulation [8,10,15]. 

The main hypotheses were that EI ability will be influenced by BMI and sleep quality, with those professionals with higher BMI or having poorer sleep quality will have poorer EI ability. However, the results obtained only allow us to affirm that as weight increases slightly, the level of emotional attention increases and the capacity for emotional repair decreases. Similarly, the longer the sleep duration, the better the emotional clarity, and the greater the daytime dysfunction, the worse the emotional repair. Therefore, the results found do not correspond to the hypotheses put forward at the beginning of the study.

In the relationship between EI and gender, no significant differences were obtained between male and female nurses. Both presented appropriate levels in the three dimensions of the scale, unlike most studies that propose that women tend to present greater attention to emotions than men, not only in this profession but at a general level [17,19,20]. This dissimilarity may be influenced by the sample size (48 women versus 14 men), so it should be taken into consideration for statistical purposes.

The findings obtained in the relationship between EI and age show us that younger nurses and/or those who have just finished their university degree have a better ability to feel and express feelings, as well as to understand emotions, in contrast to nurses with a more adult age. On the other hand, through the different tests carried out, significant data have been obtained indicating that the higher age ranges present a better capacity to regulate emotions than the lower ranges. These results are in line with those found in the study by Extremera et al. [21], which shows that age does influence the development of EI skills, improving over time. Since, as can be seen in the present study, emotion repair visibly improves from early adulthood to middle adulthood. Moreover, in the study by Navarro et al. [48] it has been observed that the older the age, the better the emotion regulation, compared to a younger age group.

Between EI and time of work experience in an emergency department, no significant data were found in the ability to feel and express feelings or in the ability to understand emotions, but significant differences were found in the ability to regulate emotional states. These differences indicated that nurses with less experience in this service have a worse ability to regulate emotions; however, this does not occur in nurses who have been working in the emergency department for many years. This is in line with the study conducted by Cook et al. [22], which shows that the EI level of accountants will be influenced by the length of work experience, i.e., a person will have a better level of EI the longer he or she has worked in a job.

Another objective of this study was to relate the EI ability of emergency nurses to their BMI. It could be seen that more than half of the sample (53%) presented normal weight, which does not correspond to what was found in the studies of Gázquez Linares et al. [24], and Kruger [35] in which it was observed that the nursing staff generally presented overweight and obesity due to poor diet. On the other hand, after correlating the data obtained through the survey, it has been seen that these professionals with grade II overweight and type I obesity are able to feel and express feelings, while other professionals with body mass indexes do not present this relationship. These data show a difference with the data exposed in the studies of Gázquez Linares et al. [24] and Salafia et al. [49], which explain that as a person’s weight increases, the level of EI decreases. Nevertheless, in the relationship between BMI and the ability to regulate emotional states, a significance is found indicating that as weight increases to type I obesity the ability to regulate emotions decreases, which is consistent with the studies of both Gázquez Linares et al. [24] and Salafia et al. [49].

The last objective set for this study was to know if there was any relationship between EI and sleep quality of nurses working in emergency departments, generally with long and exhausting work shifts. Gázquez Linares et al. [24] refers that nurses generally suffer alterations in sleep quality. The findings of the present study are in the same line as this study, since 66% of the sample presented sleep quality alterations. After correlating the data between EI and the seven components of the PSQI, significant differences were found in three of the areas, showing that the longer the duration of sleep, the better the ability to understand emotions, and the greater the daytime dysfunction, the worse the ability to regulate emotional states. Both results are consistent with the observations of Abdali et al. [28], who show that lack of sleep impairs the ability to process emotions. The last component in which significant data were found was the use of medication, in which it was seen that an increase in consumption is related to a lower ability to understand emotions, as well as Kun et al. [50], who relate a lower EI to the consumption of certain substances and treatments. Another problem observed in the study by Abdali et al. [28] and Duffy et al. [33] was a masking of the lack of emotional management in the form of nightmares, and related to these data, in this study it has been concluded that up to 64.1% of nurses working in an emergency department have nightmares a week in the last month.

There are some limitations in this study, such as the sample size, which may have limited value in some of the tests performed. Another limitation is that both the TMMS-24 and the PSQI may be subject to bias, since subjective aspects are evaluated and may present individual changes. In addition, some variables not included in the statistical analysis could have influenced the results (e.g., shiftwork and health status). Future research should take these confounding variables into account.

The results presented allow us to know the level of EI of emergency nurses and their quality of sleep, as well as the relationship with variables, such as age, gender, time of experience and BMI, which can lead to individual and/or collective reflection and make it possible to improve the health, whether mental or physical, of these professionals. 

Analyzing the influence of emotional intelligence and sleep quality in emergency nurses is of great importance because of its relationship to burnout and leaving the profession. Nurses leaving their jobs is an issue of international concern, as the demand for nurses is increasing [51]. 

Research around emotional intelligence is useful to further nursing education research and to develop a teaching approach. Educational interventions have been used primarily to improve compassionate care in this population [52,53].

Furthermore, sleep pattern interventions that positively promote nurses’ health and encourage effective work performance should be developed [54].

Based on all the results found, it is recommended that future research explore these same variables for more information in the field of emergency nursing, since there is little information related in such a concrete way, and it is requested that other variables that may be related to EI and sleep quality, such as stress and work shifts, be taken into consideration. It is also suggested to analyze which interventions are used by emergency nurses to address their psychological needs and thus maintain an appropriate level of EI.

## 5. Conclusions

This study concludes that both male and female emergency nurses have an appropriate level of EI, and no significant differences were found between genders. In addition, it has been observed that younger nurses and/or nurses who recently completed their nursing degree have a better ability to feel, express and to understand emotional states, in contrast to nurses of an older age. However, older emergency nurses have been found to have better emotional regulation and repair skills than younger nurses. 

It has been found that nurses that are overweight feel and express their feelings better. Nevertheless, type I obesity is related to professionals who regulate their emotional states more inappropriately. Finally, the quality of sleep of emergency nurses is clearly impaired. This lack of sleep impairs the ability to process and understand emotions.

## Figures and Tables

**Table 1:** General characteristics of the subjects.

		*n*	%
Gender	Female	48	77
	Male	14	23
Age	21–30 years	30	48
	31–40 years	19	31
	41–50 years	10	15
	51–60 years	3	5
Length of work experience	<1 months	6	10
	2–6 months	15	24
	7–12 months	4	6
	1–5 years	21	34
	6–10 years	6	10
	11–20 years	8	13
	>21 years	2	3

%: percentage; *n*: sample size (group).

**Table 2:** Descriptive analyses of the main variables.

	Mean	SD	Minimum Range	Maximum Range
BMI	24.18	3.87	17.43	34.29
Attention	27.05	5.29	13	37
Clarity	27.56	5.70	15	40
Repair	28.76	5.59	15	40
PSQI	7.44	3.76	1	16

BMI: body mass index; SD: standard deviation; PSQI: Pittsburgh Sleep Quality Index.

**Table 3:** Evaluation of the emotional intelligence Trait Meta-Mood Scale in emergency nurses.

	Attention	Clarity	Repair
Low (%)	29.0	29.0	19.4
Appropriate (%)	64.5	62.9	67.7
Excellent (%)	6.5	8.1	12.9

%: percentage.

**Table 4:** Pittsburgh Sleep Quality Index scale in emergency nurses.

	No Difficulty	Mild Difficulty	Moderate Difficulty	Severe Difficulty
Subjective sleep quality (%)	26	42	23	10
Sleep latency (%)	10	39	29	23
Sleep duration (%)	44	37	16	3
Habitual sleep efficiency (%)	35	37	10	18
Sleep disturbances (%)	5	65	31	0
Use of sleeping medication (%)	74	11	8	6
Daytime dysfunction (%)	32	39	26	3

%: percentage.

**Table 5:** Relationship between emotional intelligence and sex in nursing professionals working in the emergency department.

		Women	Men	*p*
Attention	Low (%)	28.6	29.2	0.860
	Appropriate (%)	71.4	62.5	
	Too much (%)	0	8.3	
Clarity	Low (%)	28.6	29.2	0.220
	Appropriate (%)	64.3	62.5	
	Too much (%)	7.1	8.3	
Repair	Low (%)	7.1	22.9	0.084
	Appropriate (%)	78.6	64.6	
	Too much (%)	14.3	12.5	

%: percentage.

**Table 6:** Pearson correlation between emotional intelligence and body mass index of emergency nurses.

	Underweight	Normal Weight	Overweight I	Overweight II	Obesity
Appropriate attention	−0.896	0.075	0.377	0.858 **	0.828 *
Inappropriate attention	0.941	−0.052	−0.109	−0.639	0.297
Appropriate clarity	0.149	0.176	−0.277	−0.491	0.242
Inappropriate clarity	0.444	0.436	0.091	0.067	−0.175
Appropriate repair	0.896	−0.115	0.380	−0.274	−0.762
Inappropriate repair	−0.064	−0.339	−0.079	−0.009	0.877 *

* *p* < 0.05; ** *p* < 0.01.

**Table 7:** Pearson correlation between emotional intelligence and Pittsburgh Sleep Quality Index of emergency nurses.

	Appropriate Attention	Inappropriate Attention	Appropriate Clarity	Inappropriate Clarity	Appropriate Repair	Inappropriate Repair
Subnjective quality of sleep	−0.067	−0.007	0.152	−0.124	−0.105	−0.066
Sleep latency	−0.229	0.240	−0.015	−0.297	−0.182	0.249
Duration of sleep	0.153	−0.110	0.329 *	0.173	0.066	0.387
Efficiency of habitual sleep	0.133	−0.083	0.241	0.108	−0.165	0.363
Sleep disturbances	0.243	0.11	−0.100	0.036	−0.010	0.462
Medication use	−0.141	0.412	−0.393 **	−0.192	0.011	0.106
Daytime dysfunction	0.072	0.162	−0.121	0.374	−0.070	−0.797 **

* *p* < 0.05; ** *p* < 0.01.

## Data Availability

The data presented in this study is available on request from the corresponding author. The data are not publicly available due to is personal health information.
